# Bis{1-[(1*H*-benzotriazol-1-yl)meth­yl]-2-methyl-1*H*-imidazole-κ*N* ^3^}dichlorido­cobalt(II)

**DOI:** 10.1107/S1600536812026712

**Published:** 2012-06-20

**Authors:** Haiyan Yang, Yinghua Li, Yaomin Zhao, Wenzhuo Li, Fang He

**Affiliations:** a School of Materials and Chemical Engineering, Zhongyuan University of Technology, Zhengzhou 450007, People’s Republic of China

## Abstract

In the title mononuclear complex, [CoCl_2_(C_11_H_11_N_5_)_2_], the Co^II^ atom is four-coordinated by two ligand N atoms and two Cl atoms in a distorted tetra­hedral geometry. In the crystal, mol­ecules are stacked through π–π inter­actions [centroid–centroid distances = 3.473 (2), 3.807 (3), 3.883 (2) and 3.676 (2) Å], forming a three-dimensional supra­molecular network.

## Related literature

For background to complexes constructed from *N*-heterocyclic ligands, see: Yang *et al.* (2009 ▶); Meng *et al.* (2009 ▶); Mu *et al.* (2011 ▶); Zhao *et al.* (2012 ▶).
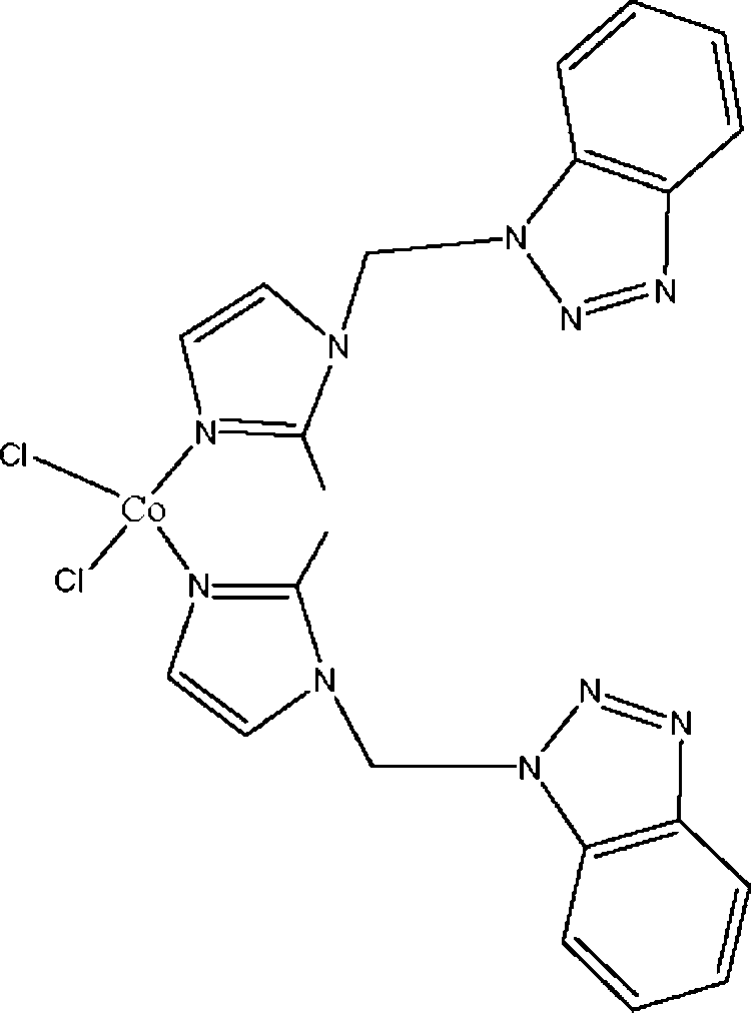


## Experimental

### 

#### Crystal data

[CoCl_2_(C_11_H_11_N_5_)_2_]
*M* *_r_* = 556.33
Triclinic, 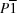

*a* = 8.1684 (16) Å
*b* = 12.691 (3) Å
*c* = 13.289 (3) Å
α = 65.48 (3)°
β = 79.66 (3)°
γ = 84.30 (3)°
*V* = 1232.5 (6) Å^3^
*Z* = 2
Mo *K*α radiation
μ = 0.95 mm^−1^
*T* = 293 K
0.22 × 0.21 × 0.18 mm

#### Data collection

Rigaku Saturn diffractometer
Absorption correction: numerical (*CrystalClear*; Rigaku/MSC, 2006 ▶) *T*_min_ = 0.819, *T*_max_ = 0.848
12593 measured reflections
4330 independent reflections
3512 reflections with *I* > 2σ(*I*)
*R*_int_ = 0.039

#### Refinement

*R*[*F* ^2^ > 2σ(*F* ^2^)] = 0.049
*wR*(*F* ^2^) = 0.101
*S* = 1.08
4330 reflections
318 parameters
H-atom parameters constrained
Δρ_max_ = 0.27 e Å^−3^
Δρ_min_ = −0.23 e Å^−3^

### 

Data collection: *CrystalClear* (Rigaku/MSC, 2006 ▶); cell refinement: *CrystalClear*; data reduction: *CrystalClear*; program(s) used to solve structure: *SHELXS97* (Sheldrick, 2008 ▶); program(s) used to refine structure: *SHELXL97* (Sheldrick, 2008 ▶); molecular graphics: *SHELXTL* (Sheldrick, 2008 ▶); software used to prepare material for publication: *SHELXTL*.

## Supplementary Material

Crystal structure: contains datablock(s) global, I. DOI: 10.1107/S1600536812026712/vm2178sup1.cif

Structure factors: contains datablock(s) I. DOI: 10.1107/S1600536812026712/vm2178Isup2.hkl

Additional supplementary materials:  crystallographic information; 3D view; checkCIF report

## Figures and Tables

**Fig. 1.**
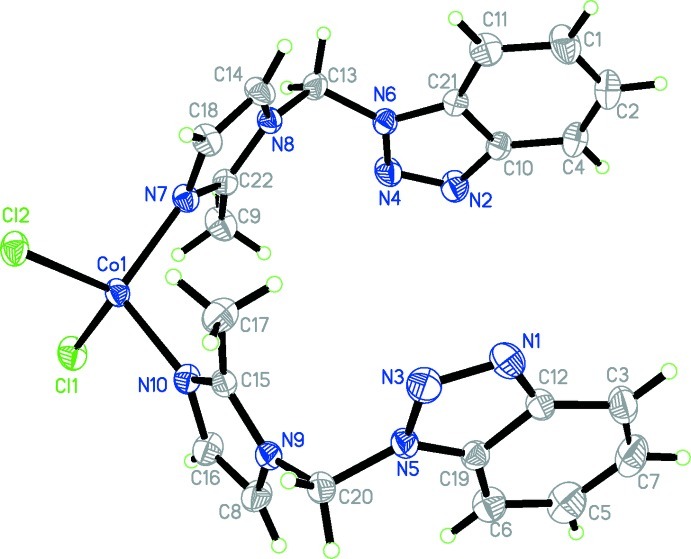
View of the title complex, showing the labeling of the 30% probability ellipsolids.

**Fig. 2.**
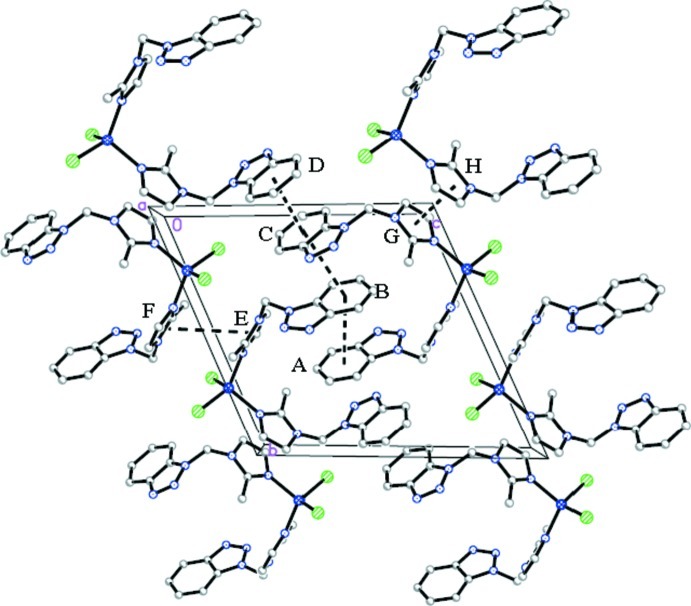
View of the title complex along the a axis, showing the π–π stacking interactions between the molecules.

## supplementary crystallographic information

### Comment

Multidentate N-heterocyclic ligands, such as imidazole, triazole, tetrazole and their derivative, have more coordination sites and can result in coordination polymers with novel network patterns (Yang *et al.*, 2009; Meng *et al.*, 2009). These ligands have been the focus of attention in coordination chemistry. In recent years, our group has designed and synthesized a series of N-heterocyclic compounds and studied their coordination behaviors (Mu *et al.*, 2011; Zhao *et al.*, 2012). As a continuation of our research, we synthesized a N-heterocyclic compound 1-[(benzotriazol-1-yl)methyl]-1-*H*-1,3-(2-methyl-imdazole), and used it as ligand to react with CoCl_2_, generating a new complex, [Co(C_11_H_11_N_5_)_2_Cl_2_] (I), which is reported here.

The crystal structure of the title compound is depicted in Fig. 1. The Co^II^ atom is four-coordinated by two N atoms from two ligands, with Co—N bond lengths of 2.012 (3) Å and 2.026 (3) Å., and two Cl atoms, with Co—Cl bond lengths of 2.2449 (10) Å and 2.2527 (14) Å. The bond angles around the Co atom vary from 102.74 (8) ° (N10—Co1—Cl1) to 115.96 (8) ° (N7—Co1—Cl1). The dihedral angle between the imidazole planes in the two ligands is 77.2 (2) °.

In the crystal structure, the adjacent mononuclear structure units are stacked on each other along the b- and *c* axis through π–π interactions as is shown in Fig. 2. The centroid-centroid distances between the two adjacent aromatic planes are 3.883 (2) Å (planes A and B), 3.807 (3) Å (planes B and C), 3.473 (2) Å (planes C and D), 4.593 (4) Å (planes E and F) and 3.676 (2) Å (planes G and H), respectively. In addition, these mononuclear structure units are parallel to each other along the *a* axis, forming a three-dimensional supramolecular network.

### Experimental

A methanol solution (5 ml) of 1-((benzotriazol-1-yl)methyl)- 1-*H*-1,3-(2-methyl-imdazol) (0.1 mmol) was added dropwise into a methanol solution (3 ml) of CoCl_2_ (0.05 mmol). The resulting solution was left at room temperature. After two weeks, good quality blue crystals were obtained from the solution and dried in air.

### Refinement

H atoms were generated geometrically, with C-H = 0.96, 0.97 and 0.93Å for methyl, methylene and aromatic H, respectively, and constrained to ride their parent atoms with U_iso_(H) = x times U_eq_(C), where x = 1.5 for methyl H and x = 1.2 for all other H atoms.

### Crystal data

**Table:**

[CoCl_2_(C_11_H_11_N_5_)_2_]	*Z* = 2
*M**_r_* = 556.33	*F*(000) = 570
Triclinic, *P*1	*D*_x_ = 1.499 Mg m^−^^3^
Hall symbol: -P 1	Mo *K*α radiation, λ = 0.71073 Å
*a* = 8.1684 (16) Å	Cell parameters from 3451 reflections
*b* = 12.691 (3) Å	θ = 2.5–29.2°
*c* = 13.289 (3) Å	µ = 0.95 mm^−^^1^
α = 65.48 (3)°	*T* = 293 K
β = 79.66 (3)°	Prism, blue
γ = 84.30 (3)°	0.22 × 0.21 × 0.18 mm
*V* = 1232.5 (6) Å^3^	

### Data collection

**Table:**

Rigaku Saturn diffractometer	4330 independent reflections
Radiation source: fine-focus sealed tube	3512 reflections with *I* > 2σ(*I*)
Graphite monochromator	*R*_int_ = 0.039
Detector resolution: 28.5714 pixels mm^-1^	θ_max_ = 25.0°, θ_min_ = 2.5°
ω scans	*h* = −9→9
Absorption correction: numerical (*CrystalClear*; Rigaku/MSC, 2006)	*k* = −15→15
*T*_min_ = 0.819, *T*_max_ = 0.848	*l* = −15→15
12593 measured reflections	

### Refinement

**Table:**

Refinement on *F*^2^	Secondary atom site location: difference Fourier map
Least-squares matrix: full	Hydrogen site location: inferred from neighbouring sites
*R*[*F*^2^ > 2σ(*F*^2^)] = 0.049	H-atom parameters constrained
*wR*(*F*^2^) = 0.101	*w* = 1/[σ^2^(*F*_o_^2^) + (0.0391*P*)^2^ + 0.5956*P*] where *P* = (*F*_o_^2^ + 2*F*_c_^2^)/3
*S* = 1.08	(Δ/σ)_max_ = 0.001
4330 reflections	Δρ_max_ = 0.27 e Å^−^^3^
318 parameters	Δρ_min_ = −0.23 e Å^−^^3^
0 restraints	Extinction correction: *SHELXL97* (Sheldrick, 2008)
Primary atom site location: structure-invariant direct methods	Extinction coefficient: 0.0016 (4)

### Special details

**Table:**

Geometry. All e.s.d.'s (except the e.s.d. in the dihedral angle between two l.s. planes) are estimated using the full covariance matrix. The cell e.s.d.'s are taken into account individually in the estimation of e.s.d.'s in distances, angles and torsion angles; correlations between e.s.d.'s in cell parameters are only used when they are defined by crystal symmetry. An approximate (isotropic) treatment of cell e.s.d.'s is used for estimating e.s.d.'s involving l.s. planes.
Refinement. Refinement of *F*^2^ against ALL reflections. The weighted *R*-factor *wR* and goodness of fit *S* are based on *F*^2^, conventional *R*-factors *R* are based on *F*, with *F* set to zero for negative *F*^2^. The threshold expression of *F*^2^ > σ(*F*^2^) is used only for calculating *R*-factors(gt) *etc*. and is not relevant to the choice of reflections for refinement. *R*-factors based on *F*^2^ are statistically about twice as large as those based on *F*, and *R*- factors based on ALL data will be even larger.

### Fractional atomic coordinates and isotropic or equivalent isotropic displacement parameters (Å^2^)

**Table:**

	*x*	*y*	*z*	*U*_iso_*/*U*_eq_	
Co1	0.74031 (5)	0.25471 (4)	1.02928 (4)	0.03397 (15)	
Cl2	0.96039 (10)	0.29359 (8)	1.08673 (7)	0.0442 (2)	
Cl1	0.54177 (11)	0.15986 (8)	1.17231 (7)	0.0499 (3)	
N10	0.8046 (3)	0.1424 (2)	0.9553 (2)	0.0355 (6)	
N9	0.9277 (3)	0.0521 (2)	0.8515 (2)	0.0361 (6)	
N8	0.5347 (3)	0.5454 (2)	0.7929 (2)	0.0347 (6)	
N7	0.6681 (3)	0.4063 (2)	0.9140 (2)	0.0356 (6)	
N6	0.3355 (3)	0.5794 (2)	0.6656 (2)	0.0366 (6)	
N5	1.0308 (3)	0.0933 (2)	0.6572 (2)	0.0355 (6)	
N4	0.1979 (4)	0.5126 (3)	0.7064 (2)	0.0506 (8)	
C22	0.5192 (4)	0.4454 (3)	0.8859 (3)	0.0341 (7)	
C21	0.3696 (4)	0.6166 (3)	0.5519 (3)	0.0351 (8)	
C20	1.0485 (4)	0.0219 (3)	0.7720 (3)	0.0402 (8)	
H20A	1.1599	0.0296	0.7836	0.048*	
H20B	1.0357	−0.0585	0.7865	0.048*	
C19	0.9193 (4)	0.0867 (3)	0.5953 (3)	0.0355 (8)	
C18	0.7811 (4)	0.4857 (3)	0.8355 (3)	0.0412 (8)	
H18	0.8957	0.4805	0.8347	0.049*	
C17	1.0626 (4)	0.2316 (3)	0.8261 (3)	0.0518 (10)	
H17A	1.0585	0.2762	0.8698	0.078*	
H17B	1.0434	0.2820	0.7517	0.078*	
H17C	1.1701	0.1939	0.8228	0.078*	
N3	1.1490 (4)	0.1713 (3)	0.5909 (3)	0.0506 (8)	
C16	0.7157 (4)	0.0454 (3)	0.9789 (3)	0.0458 (9)	
H16	0.6192	0.0222	1.0307	0.055*	
C15	0.9326 (4)	0.1432 (3)	0.8783 (3)	0.0336 (7)	
C14	0.7008 (4)	0.5706 (3)	0.7612 (3)	0.0431 (9)	
H14	0.7478	0.6343	0.7001	0.052*	
C13	0.3999 (4)	0.6178 (3)	0.7388 (3)	0.0412 (8)	
H13A	0.3096	0.6206	0.7962	0.049*	
H13B	0.4391	0.6960	0.6957	0.049*	
N2	0.1465 (4)	0.5038 (3)	0.6233 (3)	0.0545 (8)	
C12	0.9755 (4)	0.1649 (3)	0.4876 (3)	0.0429 (9)	
N1	1.1164 (4)	0.2153 (3)	0.4899 (3)	0.0561 (9)	
C11	0.4878 (4)	0.6888 (3)	0.4702 (3)	0.0516 (10)	
H11	0.5704	0.7210	0.4876	0.062*	
C10	0.2473 (4)	0.5673 (3)	0.5262 (3)	0.0409 (8)	
C9	0.3583 (4)	0.3907 (3)	0.9458 (3)	0.0548 (10)	
H9A	0.3199	0.3536	0.9047	0.082*	
H9B	0.2778	0.4489	0.9524	0.082*	
H9C	0.3727	0.3341	1.0190	0.082*	
C8	0.7901 (4)	−0.0098 (3)	0.9155 (3)	0.0477 (9)	
H8	0.7552	−0.0771	0.9150	0.057*	
C7	0.7637 (6)	0.1099 (4)	0.4233 (4)	0.0684 (13)	
H7	0.7086	0.1153	0.3658	0.082*	
C6	0.7829 (4)	0.0189 (3)	0.6201 (3)	0.0506 (9)	
H6	0.7453	−0.0331	0.6926	0.061*	
C5	0.7071 (5)	0.0326 (4)	0.5325 (4)	0.0657 (12)	
H5	0.6143	−0.0109	0.5456	0.079*	
C4	0.2367 (5)	0.5902 (4)	0.4153 (3)	0.0541 (10)	
H4	0.1549	0.5580	0.3973	0.065*	
C3	0.8964 (6)	0.1772 (4)	0.3983 (3)	0.0606 (11)	
H3	0.9330	0.2290	0.3255	0.073*	
C2	0.3513 (5)	0.6615 (4)	0.3352 (3)	0.0623 (11)	
H2	0.3476	0.6788	0.2604	0.075*	
C1	0.4746 (5)	0.7095 (4)	0.3621 (3)	0.0645 (12)	
H1	0.5510	0.7575	0.3043	0.077*	

### Atomic displacement parameters (Å^2^)

**Table:**

	*U*^11^	*U*^22^	*U*^33^	*U*^12^	*U*^13^	*U*^23^
Co1	0.0345 (3)	0.0377 (3)	0.0270 (2)	−0.00561 (19)	−0.00427 (19)	−0.0096 (2)
Cl2	0.0399 (5)	0.0547 (5)	0.0383 (5)	−0.0103 (4)	−0.0084 (4)	−0.0162 (4)
Cl1	0.0476 (5)	0.0608 (6)	0.0335 (5)	−0.0190 (4)	0.0048 (4)	−0.0121 (4)
N10	0.0339 (15)	0.0412 (16)	0.0308 (15)	−0.0087 (12)	−0.0017 (13)	−0.0135 (13)
N9	0.0444 (16)	0.0333 (15)	0.0281 (15)	−0.0064 (13)	−0.0051 (13)	−0.0090 (12)
N8	0.0347 (15)	0.0357 (15)	0.0331 (15)	−0.0004 (12)	−0.0090 (12)	−0.0121 (13)
N7	0.0327 (15)	0.0389 (16)	0.0318 (15)	−0.0045 (12)	−0.0031 (12)	−0.0109 (13)
N6	0.0350 (15)	0.0396 (16)	0.0366 (16)	−0.0028 (12)	−0.0104 (13)	−0.0144 (13)
N5	0.0402 (16)	0.0354 (15)	0.0287 (15)	−0.0065 (12)	−0.0038 (13)	−0.0102 (12)
N4	0.0496 (19)	0.056 (2)	0.0401 (18)	−0.0179 (15)	−0.0051 (15)	−0.0106 (15)
C22	0.0317 (18)	0.0379 (19)	0.0318 (18)	−0.0050 (14)	−0.0030 (15)	−0.0130 (15)
C21	0.0297 (18)	0.0391 (19)	0.0383 (19)	0.0046 (15)	−0.0070 (15)	−0.0178 (16)
C20	0.047 (2)	0.041 (2)	0.0327 (19)	0.0042 (16)	−0.0083 (16)	−0.0154 (16)
C19	0.0361 (18)	0.0365 (19)	0.0354 (19)	0.0007 (15)	−0.0055 (15)	−0.0164 (16)
C18	0.0285 (18)	0.050 (2)	0.039 (2)	−0.0075 (16)	−0.0031 (16)	−0.0114 (17)
C17	0.055 (2)	0.054 (2)	0.048 (2)	−0.0167 (19)	0.0076 (19)	−0.0263 (19)
N3	0.058 (2)	0.0488 (18)	0.0410 (18)	−0.0219 (16)	0.0027 (16)	−0.0144 (15)
C16	0.044 (2)	0.053 (2)	0.042 (2)	−0.0197 (18)	0.0044 (17)	−0.0209 (18)
C15	0.0377 (18)	0.0326 (18)	0.0294 (17)	−0.0040 (14)	−0.0092 (15)	−0.0092 (15)
C14	0.040 (2)	0.041 (2)	0.038 (2)	−0.0114 (16)	−0.0046 (16)	−0.0040 (16)
C13	0.045 (2)	0.040 (2)	0.043 (2)	0.0046 (16)	−0.0163 (17)	−0.0192 (17)
N2	0.0509 (19)	0.065 (2)	0.048 (2)	−0.0192 (16)	−0.0110 (16)	−0.0184 (17)
C12	0.055 (2)	0.038 (2)	0.033 (2)	0.0056 (17)	−0.0085 (17)	−0.0136 (16)
N1	0.074 (2)	0.0477 (19)	0.0361 (18)	−0.0151 (17)	0.0057 (16)	−0.0091 (15)
C11	0.036 (2)	0.061 (2)	0.055 (2)	−0.0104 (18)	−0.0029 (18)	−0.021 (2)
C10	0.0387 (19)	0.046 (2)	0.042 (2)	−0.0005 (16)	−0.0097 (17)	−0.0205 (17)
C9	0.036 (2)	0.065 (3)	0.048 (2)	−0.0130 (18)	−0.0003 (18)	−0.009 (2)
C8	0.058 (2)	0.046 (2)	0.040 (2)	−0.0248 (19)	0.0001 (18)	−0.0167 (18)
C7	0.074 (3)	0.084 (3)	0.073 (3)	0.040 (3)	−0.048 (3)	−0.051 (3)
C6	0.044 (2)	0.056 (2)	0.053 (2)	−0.0062 (18)	−0.0097 (19)	−0.022 (2)
C5	0.046 (2)	0.082 (3)	0.084 (3)	0.009 (2)	−0.026 (2)	−0.044 (3)
C4	0.053 (2)	0.071 (3)	0.049 (2)	0.000 (2)	−0.015 (2)	−0.032 (2)
C3	0.084 (3)	0.059 (3)	0.038 (2)	0.028 (2)	−0.020 (2)	−0.022 (2)
C2	0.065 (3)	0.085 (3)	0.043 (2)	0.002 (2)	−0.009 (2)	−0.032 (2)
C1	0.057 (3)	0.083 (3)	0.041 (2)	−0.015 (2)	0.010 (2)	−0.018 (2)

### Geometric parameters (Å, º)

**Table:**

Co1—N7	2.012 (3)	C17—H17A	0.9600
Co1—N10	2.026 (3)	C17—H17B	0.9600
Co1—Cl2	2.2449 (10)	C17—H17C	0.9600
Co1—Cl1	2.2527 (14)	N3—N1	1.289 (4)
N10—C15	1.323 (4)	C16—C8	1.339 (5)
N10—C16	1.385 (4)	C16—H16	0.9300
N9—C15	1.350 (4)	C14—H14	0.9300
N9—C8	1.372 (4)	C13—H13A	0.9700
N9—C20	1.457 (4)	C13—H13B	0.9700
N8—C22	1.351 (4)	N2—C10	1.371 (4)
N8—C14	1.375 (4)	C12—N1	1.381 (5)
N8—C13	1.452 (4)	C12—C3	1.396 (5)
N7—C22	1.324 (4)	C11—C1	1.372 (5)
N7—C18	1.387 (4)	C11—H11	0.9300
N6—N4	1.365 (4)	C10—C4	1.396 (5)
N6—C21	1.366 (4)	C9—H9A	0.9600
N6—C13	1.446 (4)	C9—H9B	0.9600
N5—N3	1.360 (4)	C9—H9C	0.9600
N5—C19	1.361 (4)	C8—H8	0.9300
N5—C20	1.441 (4)	C7—C3	1.355 (6)
N4—N2	1.298 (4)	C7—C5	1.397 (6)
C22—C9	1.480 (4)	C7—H7	0.9300
C21—C10	1.390 (4)	C6—C5	1.355 (5)
C21—C11	1.387 (5)	C6—H6	0.9300
C20—H20A	0.9700	C5—H5	0.9300
C20—H20B	0.9700	C4—C2	1.357 (5)
C19—C6	1.383 (4)	C4—H4	0.9300
C19—C12	1.386 (4)	C3—H3	0.9300
C18—C14	1.332 (5)	C2—C1	1.395 (6)
C18—H18	0.9300	C2—H2	0.9300
C17—C15	1.480 (4)	C1—H1	0.9300
			
N7—Co1—N10	108.31 (11)	N10—C15—N9	110.3 (3)
N7—Co1—Cl2	106.00 (8)	N10—C15—C17	125.8 (3)
N10—Co1—Cl2	111.09 (8)	N9—C15—C17	123.9 (3)
N7—Co1—Cl1	115.96 (8)	C18—C14—N8	106.3 (3)
N10—Co1—Cl1	102.74 (8)	C18—C14—H14	126.8
Cl2—Co1—Cl1	112.73 (4)	N8—C14—H14	126.8
C15—N10—C16	106.0 (3)	N6—C13—N8	114.5 (3)
C15—N10—Co1	129.7 (2)	N6—C13—H13A	108.6
C16—N10—Co1	124.2 (2)	N8—C13—H13A	108.6
C15—N9—C8	107.5 (3)	N6—C13—H13B	108.6
C15—N9—C20	126.7 (3)	N8—C13—H13B	108.6
C8—N9—C20	125.7 (3)	H13A—C13—H13B	107.6
C22—N8—C14	108.1 (3)	N4—N2—C10	108.7 (3)
C22—N8—C13	126.5 (3)	N1—C12—C19	108.3 (3)
C14—N8—C13	125.4 (3)	N1—C12—C3	130.9 (4)
C22—N7—C18	106.3 (3)	C19—C12—C3	120.7 (4)
C22—N7—Co1	130.8 (2)	N3—N1—C12	108.7 (3)
C18—N7—Co1	122.4 (2)	C1—C11—C21	115.5 (3)
N4—N6—C21	110.0 (3)	C1—C11—H11	122.2
N4—N6—C13	118.9 (3)	C21—C11—H11	122.2
C21—N6—C13	129.8 (3)	N2—C10—C21	108.9 (3)
N3—N5—C19	110.3 (3)	N2—C10—C4	130.2 (3)
N3—N5—C20	119.8 (3)	C21—C10—C4	120.9 (3)
C19—N5—C20	129.4 (3)	C22—C9—H9A	109.5
N2—N4—N6	108.5 (3)	C22—C9—H9B	109.5
N7—C22—N8	109.6 (3)	H9A—C9—H9B	109.5
N7—C22—C9	126.4 (3)	C22—C9—H9C	109.5
N8—C22—C9	124.0 (3)	H9A—C9—H9C	109.5
N6—C21—C10	103.9 (3)	H9B—C9—H9C	109.5
N6—C21—C11	133.7 (3)	C16—C8—N9	106.7 (3)
C10—C21—C11	122.3 (3)	C16—C8—H8	126.6
N5—C20—N9	112.9 (3)	N9—C8—H8	126.6
N5—C20—H20A	109.0	C3—C7—C5	122.1 (4)
N9—C20—H20A	109.0	C3—C7—H7	119.0
N5—C20—H20B	109.0	C5—C7—H7	119.0
N9—C20—H20B	109.0	C5—C6—C19	116.2 (4)
H20A—C20—H20B	107.8	C5—C6—H6	121.9
N5—C19—C6	133.5 (3)	C19—C6—H6	121.9
N5—C19—C12	104.1 (3)	C6—C5—C7	122.2 (4)
C6—C19—C12	122.3 (3)	C6—C5—H5	118.9
C14—C18—N7	109.7 (3)	C7—C5—H5	118.9
C14—C18—H18	125.1	C2—C4—C10	117.0 (3)
N7—C18—H18	125.1	C2—C4—H4	121.5
C15—C17—H17A	109.5	C10—C4—H4	121.5
C15—C17—H17B	109.5	C7—C3—C12	116.6 (4)
H17A—C17—H17B	109.5	C7—C3—H3	121.7
C15—C17—H17C	109.5	C12—C3—H3	121.7
H17A—C17—H17C	109.5	C4—C2—C1	121.6 (4)
H17B—C17—H17C	109.5	C4—C2—H2	119.2
N1—N3—N5	108.6 (3)	C1—C2—H2	119.2
C8—C16—N10	109.4 (3)	C11—C1—C2	122.7 (4)
C8—C16—H16	125.3	C11—C1—H1	118.7
N10—C16—H16	125.3	C2—C1—H1	118.7
			
N7—Co1—N10—C15	68.8 (3)	Co1—N10—C15—C17	−0.5 (5)
Cl2—Co1—N10—C15	−47.3 (3)	C8—N9—C15—N10	−0.4 (4)
Cl1—Co1—N10—C15	−168.0 (3)	C20—N9—C15—N10	−178.6 (3)
N7—Co1—N10—C16	−111.3 (3)	C8—N9—C15—C17	−179.4 (3)
Cl2—Co1—N10—C16	132.7 (2)	C20—N9—C15—C17	2.4 (5)
Cl1—Co1—N10—C16	11.9 (3)	N7—C18—C14—N8	0.2 (4)
N10—Co1—N7—C22	91.0 (3)	C22—N8—C14—C18	0.0 (4)
Cl2—Co1—N7—C22	−149.7 (3)	C13—N8—C14—C18	176.8 (3)
Cl1—Co1—N7—C22	−23.8 (3)	N4—N6—C13—N8	95.8 (4)
N10—Co1—N7—C18	−79.4 (3)	C21—N6—C13—N8	−98.6 (4)
Cl2—Co1—N7—C18	39.9 (3)	C22—N8—C13—N6	−81.8 (4)
Cl1—Co1—N7—C18	165.8 (2)	C14—N8—C13—N6	102.0 (4)
C21—N6—N4—N2	1.7 (4)	N6—N4—N2—C10	−1.6 (4)
C13—N6—N4—N2	169.9 (3)	N5—C19—C12—N1	−0.5 (4)
C18—N7—C22—N8	0.4 (4)	C6—C19—C12—N1	−177.7 (3)
Co1—N7—C22—N8	−171.2 (2)	N5—C19—C12—C3	176.2 (3)
C18—N7—C22—C9	−179.2 (3)	C6—C19—C12—C3	−0.9 (5)
Co1—N7—C22—C9	9.2 (5)	N5—N3—N1—C12	−0.9 (4)
C14—N8—C22—N7	−0.3 (4)	C19—C12—N1—N3	0.9 (4)
C13—N8—C22—N7	−177.0 (3)	C3—C12—N1—N3	−175.4 (4)
C14—N8—C22—C9	179.3 (3)	N6—C21—C11—C1	−176.7 (4)
C13—N8—C22—C9	2.7 (5)	C10—C21—C11—C1	0.8 (5)
N4—N6—C21—C10	−1.0 (3)	N4—N2—C10—C21	0.9 (4)
C13—N6—C21—C10	−167.6 (3)	N4—N2—C10—C4	−175.8 (4)
N4—N6—C21—C11	176.8 (4)	N6—C21—C10—N2	0.1 (4)
C13—N6—C21—C11	10.2 (6)	C11—C21—C10—N2	−178.1 (3)
N3—N5—C20—N9	110.2 (3)	N6—C21—C10—C4	177.2 (3)
C19—N5—C20—N9	−79.0 (4)	C11—C21—C10—C4	−1.0 (5)
C15—N9—C20—N5	−77.1 (4)	N10—C16—C8—N9	0.2 (4)
C8—N9—C20—N5	105.0 (4)	C15—N9—C8—C16	0.1 (4)
N3—N5—C19—C6	176.6 (4)	C20—N9—C8—C16	178.3 (3)
C20—N5—C19—C6	5.1 (6)	N5—C19—C6—C5	−175.7 (4)
N3—N5—C19—C12	0.0 (3)	C12—C19—C6—C5	0.4 (5)
C20—N5—C19—C12	−171.5 (3)	C19—C6—C5—C7	0.5 (6)
C22—N7—C18—C14	−0.4 (4)	C3—C7—C5—C6	−1.0 (7)
Co1—N7—C18—C14	172.1 (2)	N2—C10—C4—C2	176.8 (4)
C19—N5—N3—N1	0.6 (4)	C21—C10—C4—C2	0.4 (6)
C20—N5—N3—N1	173.0 (3)	C5—C7—C3—C12	0.5 (6)
C15—N10—C16—C8	−0.5 (4)	N1—C12—C3—C7	176.3 (4)
Co1—N10—C16—C8	179.6 (2)	C19—C12—C3—C7	0.4 (5)
C16—N10—C15—N9	0.5 (4)	C10—C4—C2—C1	0.3 (6)
Co1—N10—C15—N9	−179.5 (2)	C21—C11—C1—C2	−0.1 (6)
C16—N10—C15—C17	179.5 (3)	C4—C2—C1—C11	−0.4 (7)
